# Morphological analysis of ventricular septal defect by echocardiography for prediction of aortic regurgitation in pediatric population

**DOI:** 10.1038/s41598-023-32940-7

**Published:** 2023-04-24

**Authors:** Fedoua El Louali, Floriane Soler, Virginie Fouilloux, Morgane Evin, Caroline Ovaert

**Affiliations:** 1 Pediatric and Congenital Cardiology Department, La Timone Hospital, Assistance Publique – Hôpitaux de Marseille, Marseille, France; 2 Laboratory of Biomechanics and Application, UMRT24, Gustave Eiffel University, Aix Marseille University, Marseille, France; 3 Inserm U1251, Marseille Medical Genetics, Aix Marseille University, Marseille, France

**Keywords:** Anatomy, Cardiology, Risk factors

## Abstract

Ventricular septal defects (VSD) are the most common congenital heart diseases in children. Among them, perimembranous VSD (pm-VSD) have a higher risk of complications, including aortic valve prolapse and aortic regurgitation (AR). The aim of our study was to assess echocardiographic criteria associated with AR during follow-up of pm-VSD. Forty children with restrictive pm-VSD, followed-up in our unit and who underwent a workable echocardiographic evaluation between 2015 and 2019 were included and retrospectively analyzed. The propensity score was used to match 15 patients with AR to 15 patients without AR. Median age was 2.2 year [1.4–5.7]. Median weight was 14 kg [9.9–20.3]. Aortic annulus z-score, Valsalva sinus z-score, sinotubular junction z-score, valve prolapse and commissure commitment were significantly different between the two groups (*p* = 0.047, *p* = 0.001, *p* = 0.010, *p* = 0.007, *p* < 0.001 respectively). Aortic root dilatation, aortic valve prolapse and commissure commitment to a perimembranous VSD are associated to aortic regurgitation.

## Introduction

Ventricular septal defects (VSD) are the most common congenital heart diseases in children, representing 37% of congenital malformation^1^.Although the indication for closure of hemodynamically significant VSDs is no matter of debate^2^,the jury is still out regarding small, restrictive VSDs without left ventricular dilation.

In doubly committed juxta-arterial defects and perimembranous defects, the anatomical position of the defect frequently affects the mobility and function of the aortic leaflets, resulting in aortic incompetence^3^, which is generally mild at first but progressive in nature^4^. Perimembranous VSD (pm-VSD), is deemed to be less associated with AR. In this type of VSD, the mechanism of AR seems to implicate several factors.

The aim of this study was to assess echocardiographic criteria associated with aortic regurgitation in restrictive pm-VSD.

## Methods

### Study design

Between January 2015 and January 2019, a total of 158 children with pm-VSD were admitted to our department for medical or surgical treatment.

All patients < 18 year old with perimembranous VSD and for who a workable and recent (less than three months) echocardiographic evaluation was available, were considered for inclusion. Patients with non-perimembranous VSD and patients with associated congenital heart anomalies other than atrial septal defect were excluded.

**Figure 1:**
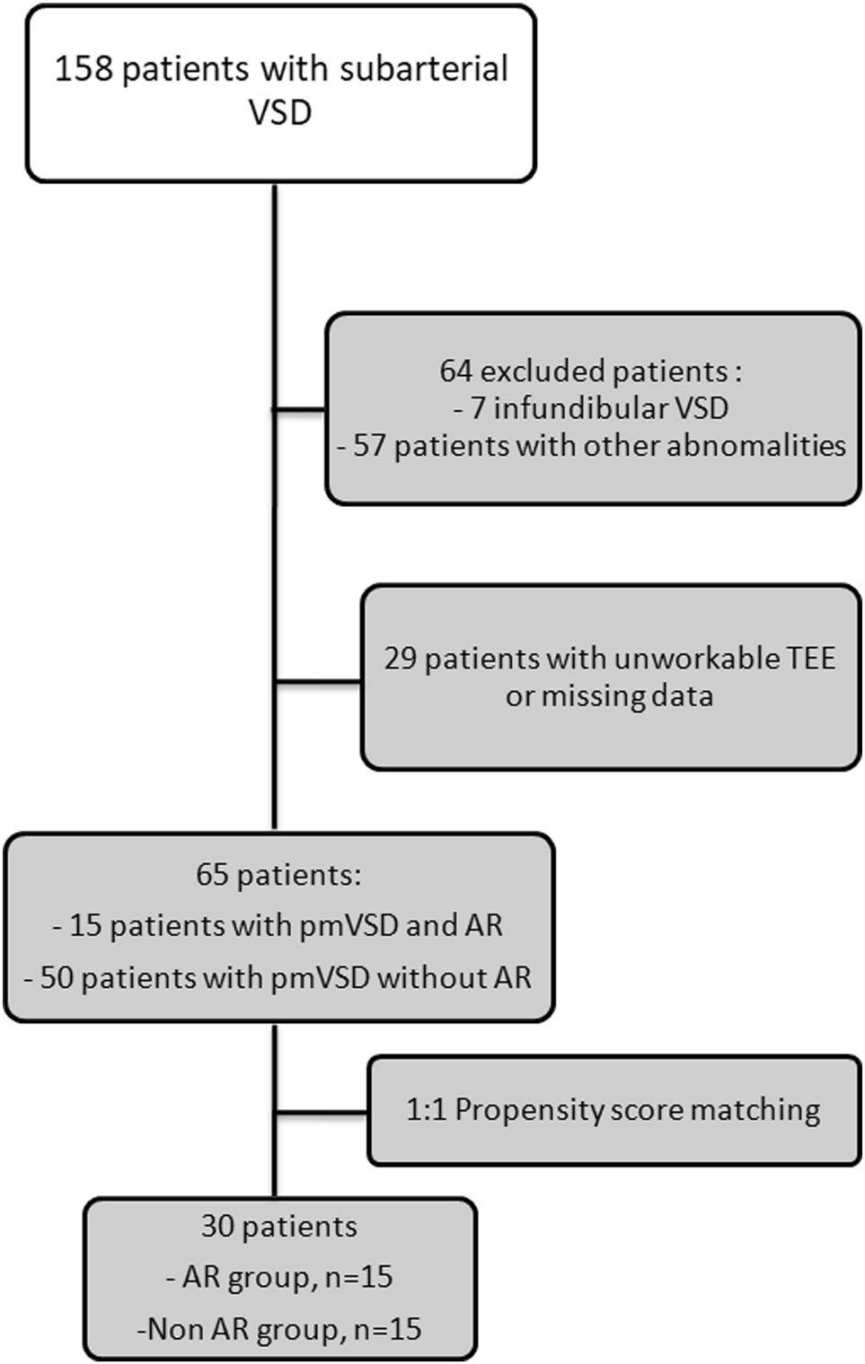
Flow-chart for the selection of the study population.

This study complies with the Declaration of Helsinki. La Timone Children’s Hospital Clinical Investigation Committee (*Direction de Recherche en Santé*) and the local ethic committee (*Commission d'Accès aux Données de Santé du CHU*) approved the study protocol (*PADS22-135*). Local ethical approval was given with waiver of informed consent for retrospective, anonymized data. Sixty five patients were eligible and thirty children were included after propensity score matching (Fig. 1: flow chart).

The cohort was divided into 2 groups:

- AR group: patient with aortic valve regurgitation (n = 15)
- Non-AR group: patient without AR (n = 15)

### Clinical data

Demographic, clinical and TTE data were collected from each patient’s medical record. The clinical parameters collected were those recorded on the day of the echocardiography.

### Echocardiographic data

All the echocardiographic studies were analyzed by the same observer (FS).

Images analyzing the VSD in 2D, color Doppler and continuous Doppler were reviewed. The size of the pm- VSD and the maximum flow velocity through the defect were recorded.

**Figure 2:**
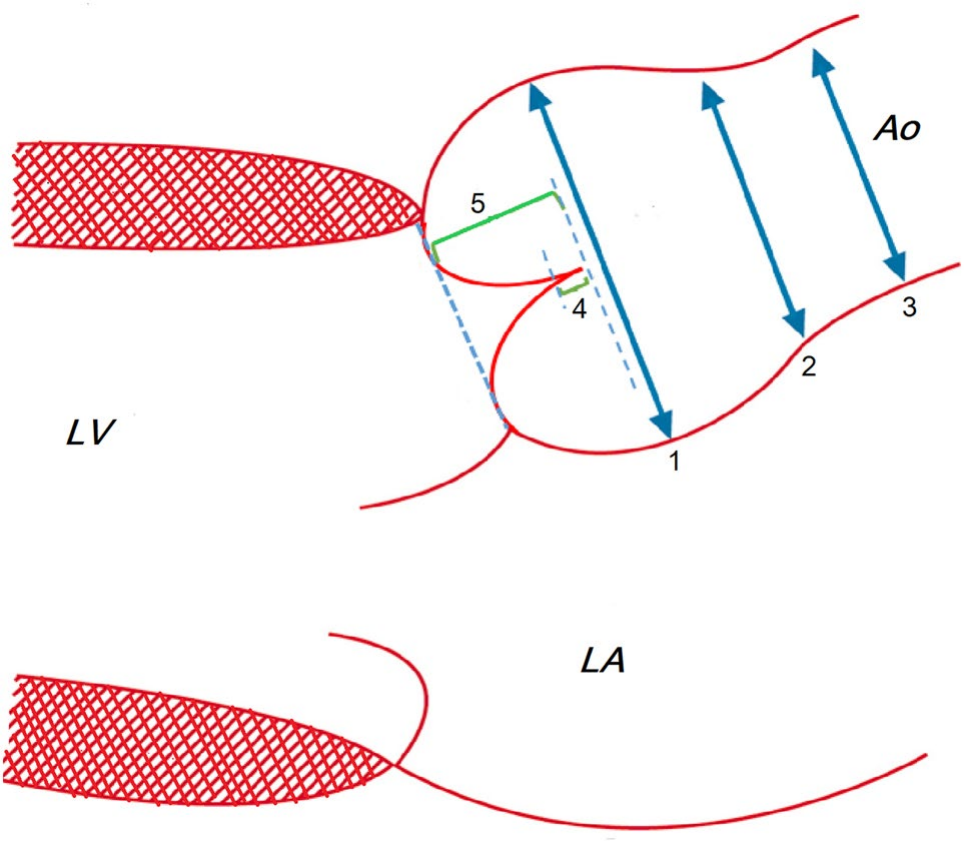
Aortic root diameters measured in 2-dimensional echocardiography: sinuses of Valsalva (1), sinotubular junction (2), and ascending aorta (3), Coaptation height (4), Effective Height (5). Ao = aorta; LA = left atrium; LV = left ventricle.

Parasternal long axis, short axis and four chamber views of the aortic valve were analyzed. Data collection included diameter of the aortic valve annulus, the Valsalva sinus and the ascending aorta (Fig. 2). Respective z-scores (ZS) were calculated according to the Detroit Formula^5^. The ratio between the VSD diameter measured in parasternal short axis (mm) and the aortic annulus diameter measured in parasternal long axis (mm) was calculated. The anatomy of the aortic valve (AV) was described and each leaflet size was measured.The cusp imbalance index was defined as: [width of right (R) or non- (N) coronary cusp/width of left coronary cusp (L)]^6^.

The coaptation height (CH) and the effective height (EH) were measured (Fig. 2). The aortic coaptation height (ACH = CH/AV ratio) and the aortic effective height (AEH = EH/AV ratio) indices were calculated as follow: ACH index% = (CH/AV) × 100, AEH index % = (EH/AV) × 100^7^.

**Figure 3:**
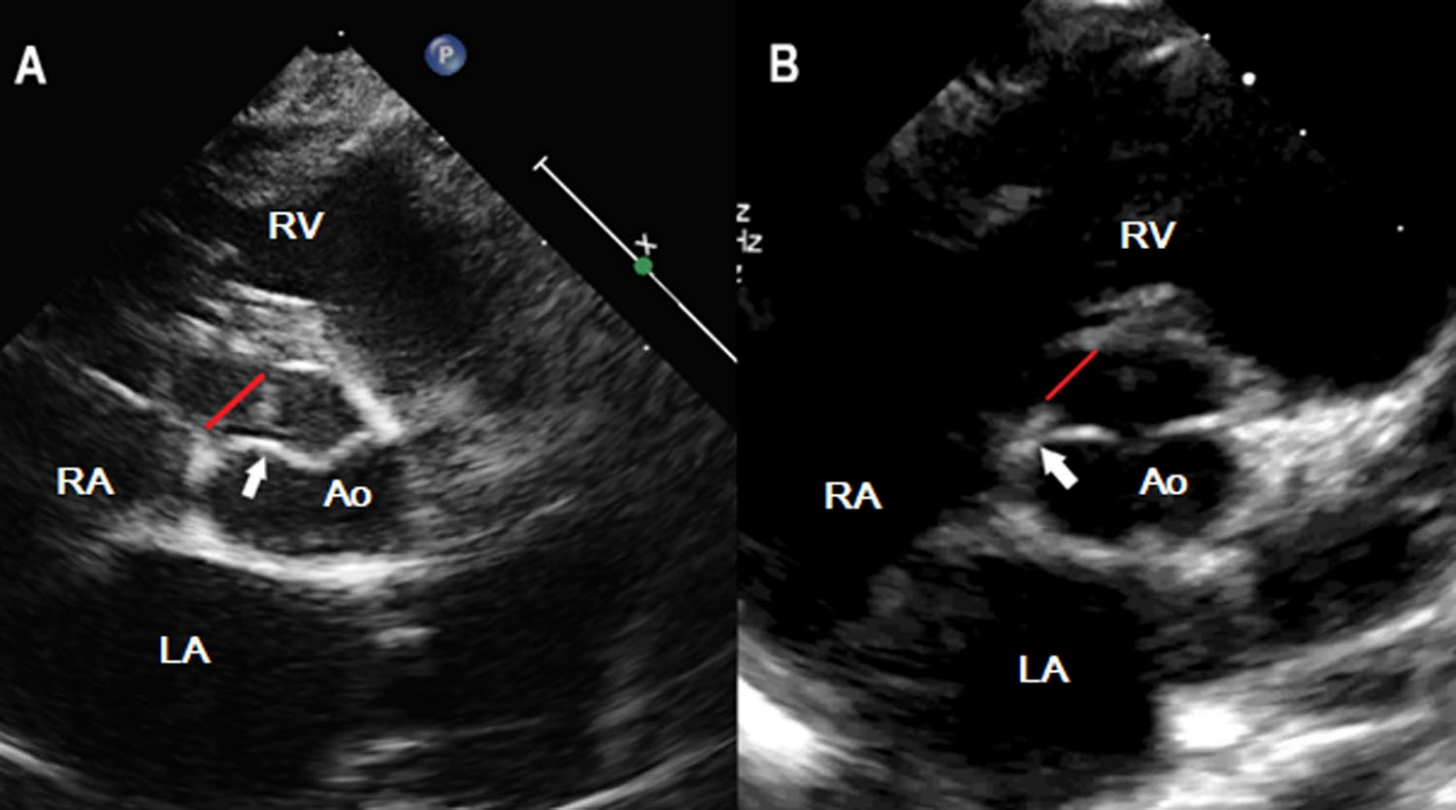
Short axis echographic views showing perimembranous ventricular septal defect (red line), A: with commissural commitment in the VSD (white arrow) and B: without commissural commitment in the VSD (white arrow). Ao = aorta; LA = left atrium; RA = right atrium; RV = right ventricle; Ao = Aorta.

Long axis and 5 chamber views were used to assess cusp (leaflet) prolapse. Commissure commitment was assessed in short axis view (Fig. 3).

### Statistical analysis

Propensity score was performed to match pm-VSD with AR patients (AR group) with pm-VSD without AR patients (Non AR group) according to age and weight. Patients were 1:1 matched using the nearest neighbour method without replacement and with a calliper of width equal to 0.25. Table 1 detailed those characteristics before and after matching.

**Table 1:** Age and weight profil of the two groups (AR group vs Non AR group) before and after matching.

	Before matching			After matching		
	AR group n = 15	Non AR group n = 50	*p*	AR group n = 15	Non AR group n = 15	*p*
Age	2 [1.2–6]	0.6 [0.3–1.7]	0.001	2.1 [1.25–6]	2.9 [1.5–6.2]	0.45
Weight	14 [9.8–22.1]	6.7 [4.5–10.6]	0.002	14 [9.8–22.1]	14 [9.9–23.1]	0.54

Continuous variables are expressed as mean (with standard deviation) or median (with IQR), where appropriate. Discrete or binary variables are presented as number (percent).

The χ^2^ test or Fisher’s exact test (if appropriate) were used for categorical variables and Mann Whitney test was used for continuous variables to assessed influencing factors Associations between echocardiographic parameters and AR were determined with univariate binary logistic regression analysis.

All p values were bilateral and significance was pronounced for a *p* value of 5%. Statistical analysis was performed using SPSS software, version 22.0 (SPSS, Inc., Chicago, IL, USA).

## Results

### Population characteristics

The median age was 2.2 year [1.4–5.7] and the median weight 14 kg [9.9–20.3]. There was no significant difference concerning demographic parameters between the two groups (Table 2).

**Table 2:** Comparison of main demographic and echographic parameters in Univariate Analysis.

	Non AR group n = 15	AR group n = 15	*P*
Male gender, n (%)	6 (40%)	11(73%)	0.057
Age, median [IQR]	2.9 [1.5–6.2]	2.1 [1.25–6]	0.45
weight, median [IQR]	14 [9.9–23.1]	14 [9.8–22.1]	0.54
ZS sinotubular junction diameter, median [IQR]	− **0.70 [**− **1.2 to **− **0.25]**	**1.6 [**− **0.70 to 2.6]**	**0.010**
ZS Valsalva sinus, median [IQR]	**0.45 [0.07–1.3]**	**3.4 [1.4–3.8]**	**0.001**
Zs aortic annulus, median [IQR]	0.95 [0–1.5]	3.5 [0.1–4.3]	0.047
ZS of end diastolic LV diameter, median [IQR]	1.2 [− 0.15 to 2.3]	1.6 [0.5–2.52]	0.607
VSD diameter, median [IQR]	6 [4–7]	6 [4–8]	0.883
VSD/Ao ratio, median [IQR]	0.40 [0.29–0.58]	0.44 [0.33–0.50]	0.868
VSD Vmax(m/s), median [IQR]	4.5 [4, 5]	4.2 [4.1–4.4]	0.262
ACH index, median [IQR]	28.5 [21.4–31.4]	31.2 [26.4–34.4]	0.116
AEH index, median [IQR]	40 [34–50]	50.0 [39.3–58.6]	0.115
The cusp imbalance index, mean ± SD	1.37 ± 0.19	1.33 ± 0.12	0.451
AV Prolapse, n (%)	**1 (6.7%)**	**8 (53.3%)**	**0.007**
Commissural commitment in VSD, n (%)	**1(6.7%)**	**10 (66.7%)**	** < 0.001**

*ZS* z score, *JST* sinotubular junction, *VSD* ventricular septal defect, *Ao* aorta, *ACH index* aortic coaptation height index, *AEH index* Aortic effective height index, *AV* aortic valve

Significant values are in bold.

Image quality was considered interpretable for all selected patients allowing successful collection of all echocardiographic data. The mean VSD diameter and VSD-to-aortic diameter ratio were respectively 6.1 ± 2.4 mm and 0.43 ± 0.13. The mean VSD peak velocity reached 4.4 ± 0.5 m/s. Median end diastolic LV diameter z score was 1.6 [0.0–2.3], 8 (26.7%) patients had a LV z-score above + 2. In this study, 25 (83.3%) patients presents restrictive VSD.

Review of previous echocardiograms of patients in the AR group shows that: two patients had a stable minimal AR. Three patients had mild to moderate AR of recent worsening and the other patients had mild AR of recent onset. In AR group, 13 (86%) patients were operated on within a year and the procedure was VSD closure only. In non AR group, 8 (53%) were operated. In the last follow up, non-operated patients are stable without AR (or AR worsening).

### Univariate analysis

Several echocardiographic parameters differed significantly between the 2 groups: (Table 2).

- In the AR group, the size of the aortic root, especially the median Valsalva sinus size, was significantly larger than in the non-AR group (3.4 [1.4–3.8] vs 0.45 [0.07–1.3], *p* = 0.001).
- In AR group, there was significantly more AVP and commissure commitment (*p* = 0.007 and *p* < 0.001 respectively).
- No significant difference was found for the AEH index, ACH index and cusp imbalance index (*p* = 0.116, *p* = 0.115, *p* = 0.451, respectively).
- The association of those parameters (aortic root size, AVP and commissure commitment) and AR is shown in Table 3.

**Table 3:** Univariate logistic regression to identify AR related factors in pm-VSD.

Variables	OR	IC (95%)	*p*
AV prolapse	16.0	[1.65–154.59]	0.017
Commissural commitment	**28.0**	**[2.82–277.9]**	**0.004**
Aortic annulus Zscore	1.53	[0.98–2.39]	0.060
Valsalva sinus Zscore	2.42	[1.29–2.53]	0.006
Sinotubular junction Zscore	2.02	[1.12–3.63]	0.019

*Pm-VSD* perimembranous ventricular septal defect, *AV* aortic valve.

Significant values are in bold.

## Discussion

Complications of *subarterial VSDs* (corresponding to doubly committed juxta arterial and perimembranous VSDs^3^), and in particular AVP have been widely studied. The incidence of AVP, reaches 73% in some studies, with progression to AR in 52 to 78% of the patients^8–13^.

Several mechanisms have been are suggested to explain the association of pm-VSD and aortic valve regurgitation. Beside aortic valve prolapse, which remains the main cause, dilatation of the ascending aorta, decrease of the coaptation height and aortic cusp imbalance have been involved.

### Dilation of the ascending aorta

Momma et al. showed that dilation of the ascending aorta was often associated with aortic valve distortion^14^. In our study, dilatation of the ascending aorta was associated with AR.

### Coaptation height

Iwashima et al., suggested a decreased coaptation height as a non-invasive marker for the assessment of severity of AVP in the presence of a VSD^7^.We used, as recommended in their publication^7^, a ratio to avoid variations in relation with patient’s weight and height difference. We did not find any statistically significant difference between the two groups.

### Aortic cusp imbalance

Tomita et al., showed that an imbalance in the width of a cusp could predict a possible progressive worsening of AR^6^.In the study of Salih and al, concerning 41 consecutive patients, operated for VSD (36 pm-VSD) with prolapsed AV, with or without AR, the presence of cusp imbalance did not impact post-operative AR improvement^15^.

In our study, this parameter was not significantly different between the two groups.

### Prolapse of aortic valve

Several studies have shown that the presence of AVP should indicate regular ultrasound monitoring for AR, which appears in more than half of the cases^16,17^. AR has a tendency to worsen as soon as it appears and may not disappear in post-operative course. Even minor prolapse can cause AR^18^ and eccentric AR can reveal AVP of the right coronary cusp that is very likely to progress^19^. In our population, AVP was found to be associated with AR.

### Commissural commitment

Our study is the first to show a significant association between the commissure commitment into the VSD and the AR. Azcarate et al.^4^ proposed a differentiation of the mechanism of aortic regurgitation according to the position of defect. In *infundibular defect* (corresponding to Outlet defect with hypoplastic or absent muscular outlet septum^3^), the mechanism seems to be a lack of support of the leaflet resulting in a gradual prolapse through the defect^4^. In pm-VSDs, infundibular septum is conserved but the defect is potentially located in the intercommissural zone of the right coronary leaflet and non-coronary leaflet (corresponding to interleaflet triangle) and incriminates both right and non-coronary leaflet^20,21^. This hypothesis is corroborated by recent publications on the relationship of the septum with the aortic valve in the context of assessment and management of transcatheter implantation of the aortic valve^22^. Indeed, due to variations in rotation of aortic root, the membranous septum can have variable relationship with its components^22,23^. Membranous septum can be located below aortic annulus, it can crossed the annulus projecting into interleaflet triangle or it can projected into the noncoronary sinus deep to the aortic leaflet^22^.

In the case where the membranous septum is located into interleaflet triangle and pm-VSD, aortic incompetence is located in the commissure due to poor coaptation of the two leaflet margins (non-coronary and right coronary leaflet)^4^. Our findings support this mechanism.

## Limitations of the study

Our study has 2 main limitations. : First of all, a technical limitation since echocardiography is a highly operator**-**dependent and observer-dependent procedure. The operator-dependency has been limited by eliminating all non-workable and incomplete ultrasound studies. The observer-dependency has been limited by proceeding to a methodical rereading of all ultrasound examinations by one single observer.

The second limitation is statistical related to the small size of our population and the retrospective nature of the study. This work, however, provides a basis for a prospective pilot study to further investigate echocardiographic parameters association with AR.

## Conclusion

**Figure 4:**
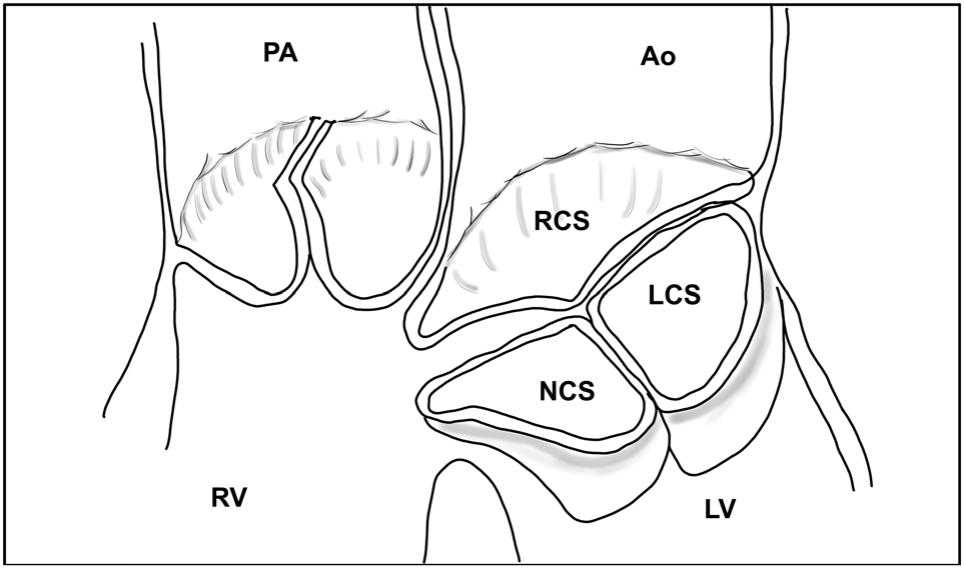
Illustration showing mechanism of aortic regurgitation through commissural commitment into VSD. Ao = aorta; LV = left ventricle; RV = right ventricle; PA = pulmonary artery; NCS = non coronary sinus; LCS = left coronary sinus; RCS = right coronary sinus.

Increased Valsalva sinus size as well as aortic valve prolapse and commissural commitment are clearly implicated and associated with AR in pm-VSD. Our study reinforces the idea that anatomical variables are directly implicated in AR. We hypothesize that commissural commitment to the VSD (Fig. 4) must be considered as a risk factor for aortic regurgitation. Further prospective studies are necessary to validate these preliminary data.

## Data Availability

Data generated during the current study are available from the corresponding author on reasonable request.
